# The Development of the Problematic Online Gaming Questionnaire (POGQ)

**DOI:** 10.1371/journal.pone.0036417

**Published:** 2012-05-10

**Authors:** Zsolt Demetrovics, Róbert Urbán, Katalin Nagygyörgy, Judit Farkas, Mark D. Griffiths, Orsolya Pápay, Gyöngyi Kökönyei, Katalin Felvinczi, Attila Oláh

**Affiliations:** 1 Eötvös Loránd University, Institute of Psychology, Budapest, Hungary; 2 Nottingham Trent University, Psychology Division, Nottingham, United Kingdom; 3 Doctoral School of Psychology, Eötvös Loránd University, Budapest, Hungary; Federal University of Rio de Janeiro, Brazil

## Abstract

**Background:**

Online gaming has become increasingly popular. However, this has led to concerns that these games might induce serious problems and/or lead to dependence for a minority of players. Aim: The aim of this study was to uncover and operationalize the components of problematic online gaming.

**Methods:**

A total of 3415 gamers (90% males; mean age 21 years), were recruited through online gaming websites. A combined method of exploratory factor analysis (EFA) and confirmatory factor analysis (CFA) was applied. Latent profile analysis was applied to identify persons at-risk.

**Results:**

EFA revealed a six-factor structure in the background of problematic online gaming that was also confirmed by a CFA. For the assessment of the identified six dimensions – preoccupation, overuse, immersion, social isolation, interpersonal conflicts, and withdrawal – the 18-item Problematic Online Gaming Questionnaire (POGQ) proved to be exceedingly suitable. Based on the latent profile analysis, 3.4% of the gamer population was considered to be at high risk, while another 15.2% was moderately problematic.

**Conclusions:**

The POGQ seems to be an adequate measurement tool for the differentiated assessment of gaming related problems on six subscales.

## Abstract (key points)

This kind of general spectrum questionnaire was born from two early initiatives [11,20]. However, psychometric characteristics of these tools were revealed only to a very limited extent. Research exploring problems in connection with video games [12,21], either offline or online, can be regarded as related attempts. Characteristics of these games and the gamer populations differ greatly from those of Massively Multiplayer Online Games (MMOG) [22]. It is therefore both essential and necessary to develop a questionnaire that (i) measures problems related to these games, (ii) is differentiated from offline games, (iii) covers all online games, and (iv) has adequate psychometric characteristics.

## Introduction

The popularity of online gaming has spread at an increasing rate since its introduction [1]. However, given the increasing number of studies on the phenomenon of problematic gaming [2], [3], [4], [5], [6], there have been growing concerns about negative consequences for a small minority. These gamers spend more time with gaming than planned while ignoring other important activities causing negative effects on their performance [4], [7], social relationships [8], [9], and withdrawal symptoms [10], [11]. There are different names present regarding this phenomenon in scientific literature. The phenomenon is referred to as addiction [11], [12], problematic use [13], [14], excessive game use [10] or engagement [15]. However, all these authors – irrespective of the name – agree in that there exists an excessive form of online gaming that shows a problematic pattern and that is related to behavioral addictions [16], [17]. The present authors propose to use the name problematic gaming. This term describes both the quintessence of the phenomenon (i.e., that the behavior is not only excessive but gaming-related problems are also expected to be present), while avoiding the notion of dependency (as the exact definition and diagnostic criteria have not yet been clarified or agreed).

The issues outlined above also suggest that precise assessment and screening of the phenomenon is an urgent matter. In order to measure the problematic nature of gaming, some authors have developed questionnaires based on the general phenomenon of internet addiction [11], while others have attempted to operationalize the behavioral addiction model of Griffiths [16], [18]. However, the limitation of these current questionnaires is that many of them typically target users of Massively Multiplayer Online Role-Playing Games (MMORPG) [4], [9], [13], [14], [19]. Though this type of game is the most popular among online games, the total population of gamers is more diverse and it is therefore necessary to develop a measure that is suitable for the assessment of other genres and gamer populations such as those who play online Real-Time Strategy (RTS) games and online First Person Shooter (FPS) games.

The aim of our study was therefore twofold. Firstly, to explore what components comprise problematic online gaming. Secondly, to make these dimensions measurable, to develop such a scale on which the identified dimensions can be assessed. Contrary to earlier studies, the objective here was to create a questionnaire suitable for all types of Massively Multiplayer Online Games. A further intention was to carry out an empirical based analysis to ensure that all components of problematic gaming remain in focus.

## Methods

The study was approved by the Institutional Review Board of the Eötvös Loránd University. Informed consent was obtained via our online system from all subjects. After introducing the goals of the study in details the subjects were asked to tick into a box if they agreed to continue and participate in the study.

### Sample, Procedure and Participants

All Hungarian websites that facilitate the playing online games were identified (n = 18). All 18 sites were contacted by the research team and were asked information about the number of visitors, and requested their cooperation in the planned study. All sites responded. Based on this information, the number of (ever) registered users was estimated to be approximately 30,000. However, many of these users may have simultaneously registered on multiple sites. Furthermore, it is likely that many formerly registered users were not currently active. All sites agreed to publish a call for participation in the present study on their home sites or via a newsletter. In the call for participation, gamers were asked to visit the study website, to sign in with a password provided by the researchers, and to complete a questionnaire. A total of 4390 questionnaires were started but not everyone completed the whole survey. This left 3415 completed questionnaires. In addition to answering the general questions regarding online gaming habits, the respondents were asked about online gaming problems.

### Measures

Major socio-demographic characteristics of the gamers (gender, age, qualification, marital status, school, work) and characteristics regarding their online gaming activities were recorded. Additionally, the survey contained a 26-item questionnaire that listed several problems regarding online gaming. The 26 items were created by means of (i) a comprehensive literature review supplemented with (ii) interviews with online gamers. Firstly, in relation to the literature review, a full search was carried out in the databases Web of Science, Science Direct, PsycINFO, and Medline using the following keywords: online gam*, MMO, MMORPG, multiplayer, FPS, First Person Shooter, RTS, Real Time Strategy. A total of 199 hits were found. However, 115 was excluded because they were irrelevant regarding specific aspects and characteristics of online gaming. The remaining 84 papers were read carefully and all items were listed out that could be considered as reflecting problematic aspects of online gaming. A total of 42 characteristics were identified this way. Secondly (and concurrently with the literature review), 15 online gamers were asked to list problems they had noticed in themselves and/or others as result of online gaming. These gamers listed 32 problems. Following exclusion of duplicates and similar items, the list was reduced to 26 items.

### Statistical Analysis

Statistical analysis comprised an exploratory factor analysis (EFA) with robust maximum-likelihood estimation (MLR) in MPLUS 6.0. The goodness of fit was assessed by the root-mean-square error of approximation (RMSEA) and its 90% confidence interval (90% CI), and p value smaller than 0.05 for test of close fit (Cfit>.05). The factor solution was selected based on fit statistics and interpretability of factors.

The factor structure based on EFA was confirmed through confirmatory factor analyses (CFA) with independent samples. CFA was performed with robust maximum-likelihood estimation (MLR) in MPLUS 6.0. The goodness of fit was evaluated using RMSEA and its 90% confidence interval (90% CI), p value smaller than 0.05 for test of close fit, standardized root-mean-square residual (SRMR), comparative fit index (CFI), and Tucker-Lewis Fit Index (TLI). As Brown (2006) and Kline (2005) recommended, multiple indices were selected in order to provide different information for evaluating model fit.

To carry out the above analyses, four non-overlapping groups from the sample were randomly selected. Sample 1 (n = 600) was used to perform an initial EFA on the original 26 items. Sample 2 (n = 600) was used to conduct a separate EFA to cross-validate the factor structure found in the first analysis. Sample 3 (n = 600) was used to conduct CFA analysis. After the inspection of modification indices, we also cross-validated the final CFA model with sample 4 (n = 1615).

In order to identify the groups of users with high risk of problematic use of online gaming a person-oriented statistical framework was selected, seeking subtypes of gamers that exhibited similar patterns of symptoms of problematic use. Therefore a latent profile analysis was performed with 1 to 6 classes with the full sample (n = 3415). The latent profile analysis [23], [24] is a latent variable analysis with a categorical latent variable – in this case problematic gamers – and continuous manifest indicators such as factor scores of POGQ. In the process of determining the number of latent classes, the Bayesian information criteria parsimony index was used, alongside the minimization of cross-classification probabilities, entropy and the interpretability of clusters. In the final determination of the number of classes, the likelihood-ratio difference test (Lo-Mendell-Rubin Adjusted LRT Test) was also used. This compares the estimated model with a model having one less class than the estimated model [25]. A low p value (<.05) indicates that the model with one less class is rejected in favor of the estimated model.

To determine the cut-off point for POGQ a sensitivity analysis based on membership in the most problematic group in the latent profile analysis was carried out. Considering membership in this group as a gold standard, the sensitivity and specificity values for all POGQ cut-off points was calculated. Thus, the accuracy of the POGQ by calculating the proportion of participants classified as being at high risk for problematic gaming versus other gamers could be assessed. The sensitivity (i.e., the proportion of true positives belonging to the most problematic group based on LPA) and specificity (i.e., the proportion of true negatives) was defined as suggested by Altman and Bland [26] and Glaros and Kline [27]. In order to explore the probability that the POGQ would give the correct “diagnosis”, the positive predictive values (PPV), the negative predictive values (NPV), and the accuracy values for each possible POGQ cut-off points was calculated. PPV was defined as the proportion of participants with positive test results who are correctly diagnosed [27], [28]. The NPV was defined as the proportion of patients with negative test results who are correctly diagnosed [27], [28].

## Results

### Descriptive Statistics

90% of our sample (n = 3072) was male. Mean age was 21 years (SD = 5.85 years). Slightly more than one-tenth of the participants had graduate education, while 39.4% had secondary education. The majority (61.9%) were primarily students, but approximately one-quarter worked full time (24.3%). Almost two-thirds of the participants were single (64.9%), and a further 24.3% were in a relationship but did not live with their partner (see Table 1).

**Table 1:** Demographics and gaming characteristics.

	Total sample (N = 3415)
Demographics	
Age, years; Mean (SD)	21.01 (5.85)
Gender (Males, %)	90
Education	
Less than high school (%)	49.5
High school graduate (%)	39.4
College graduate or more (%)	11.1
Employment	
Employed fulltime (%)	24.3
Employed part-time (%)	10.4
Student (%)	61.9
Neither employed nor student (%)	3.4
Place of residence	
Budapest (%)	26.9
Other cities (%)	54.0
Village (%)	19.1
Marital status	
Single (%)	64.9
In relationship but not living together (%)	22.3
In relationship and living together (%)	7.3
Married (%)	4.9
Divorced or widowed (%)	1.0
Subjective economic status	
Better than average (%)	47.9
Average (%)	41.8
Less than average (%)	10.3
Gaming related characteristics	
Time spent with gaming	
Less than 7 hours a week (%)	11.8
Between 7–14 hrs a week (%)	23.9
15–28 hrs a week (%)	34.8
29–42 hrs a week (%)	20.2
More than 42 hrs a week (%)	9.3
Money spent on gaming	
None (%)	51.8
Maximum 25 USD per month) (%)	36.8
More than 25 USD per month (%)	11.4
Type of players – level of organization	
Individual (%))	38.6
Amateur (%)	19.8
Semi-professional (%)	26.1
Professional (%)	15.5

Slightly more than one-third (35.7%) played a maximum 14 hours per week and approximately the same number played between 15 and 28 hours per week. One in ten played more than 42 hours a week (6 hours per day on average) (Table 1). Approximately a half spent money on gaming, although most did not spend more than $25(US) per month. The majority of the sample participants were individual gamers (38.6%) but there was also a relatively high ratio of different level organized gamers (Table 1).

### Exploratory Factor Analyses

An exploratory factor analysis was performed with maximum-likelihood estimation which is robust to non-normality and promax rotation to evaluate the factor structure of 26 items on Sample 1 (n = 600). Acceptability of the factor solution was based on goodness of fit index (RMSEA <0.08, Cfit (90% CI) <0.08, pclose >0.05), the interpretability of the solution and salient factor loadings (>0.30). A total of 1–8 factor solutions was examined. The six-factor solution provided the first adequate RMSEA value based on the criteria (χ^2^ = 409.8, df = 184 p<0.0001; RMSEA = 0.045 [0.039–0.051] pclose >0.90). The exploratory factor analysis on Sample 2 (n = 600) was repeated. As in Sample 1, a six-factor solution also provided the first adequate and interpretable factor solution (χ2 = 457.7 df = 184 p<0.0001; RMSEA = 0.050 [0.044–0.056] Cfit = 0.514). Factor loadings are presented in Table 2.

**Table 2:** Exploratory factor analyses of the generated items.

	Factor 1		Factor 2		Factor 3		Factor 4		Factor 5		Factor 6	
	1	2	1	2	1	2	1	2	1	2	1	2
Y1 When you are not gaming, how often do you think about playing a game orthink about how would it feel to play at that moment?	**0.89**	**0.93**	0.05	−0.01	−0.01	−0.02	−0.04	0.02	−0.01	0.01	0.06	−0.03
Y4 How often do you daydream about gaming?	**0.82**	**0.82**	0.03	0.07	−0.05	−0.08	0.04	−0.01	−0.01	0.05	0.09	0.09
*Y16 How often do you dream about gaming?*	***0.30***	*0.28*	*−0.13*	*−0.19*	*0.15*	*0.19*	*0.27*	*0.26*	*0.04*	*0.10*	*0.04*	*0.10*
Y3 How often do you feel that youshould reduce the amount of time you spend gaming?	0.10	0.02	**0.89**	**0.85**	0.00	0.06	−0.07	−0.07	−0.07	−0.01	−0.12	−0.21
Y6 How often do you unsuccessfully try to reduce the time you spend on gaming?	0.01	0.07	**0.87**	**0.84**	0.10	0.06	−0.05	0.03	−0.24	−0.23	0.09	0.06
Y12 How often do you feel that gaming causes problems for you in your life?	−0.07	−0.09	**0.47**	**0.63**	−0.10	−0.07	0.19	0.05	0.23	0.15	0.03	0.13
*Y11 How often do you neglect your* *studies, work or other important duties because of your gaming?*	*0–04*	*−0.07*	***0.39***	***0.38***	*0.11*	*0.26*	*0.12*	*−0.01*	*0.15*	*0.18*	*−0.03*	*0.10*
*Y2 How often do you neglect your tasks at home in order to play games more?*	*0.26*	*0.21*	***0.30***	***0.38***	*0.21*	*0.27*	*0.03*	*−0.03*	*0.10*	*0.09*	*0.01*	*−0.06*
*Y29 How often do you think about getting professional help to reduce your time* *spent gaming?*	*−0.17*	*−0.16*	***0.30***	***0.40***	*−0.03*	*−0.19*	*0.23*	*0.21*	*0.25*	*0.11*	*0.01*	*0.25*
*Y9 How often do you try to keep the amount of time you spent on gaming secret* *from people around you?*	*−0.13*	*0.01*	***0.40***	*0.21*	*0.03*	*0.19*	*−0.06*	*0.03*	*0.18*	*0.26*	*0.19*	*0.09*
Y20 How often do you lose track of time when gaming?	−0.05	−0.04	0.04	0.04	**0.75**	**0.76**	−0.04	0.00	0.01	−0.03	−0.06	−0.03
Y23 How often do you play longer than originally planned?	−0.04	−0.08	0.09	0.10	**0.69**	**0.70**	−0.05	0.08	0.10	−0.08	−0.06	0.01
Y28 How often do you feel time stops while gaming?	−0.09	−0.02	0.02	−0.07	**0.69**	**0.62**	0.05	−0.01	−0.17	−0.04	0.04	0.18
Y22 How often are you so immersed in gaming that you forget to eat?	−0.05	−0.08	−0.06	−0.09	**0.61**	**0.71**	0.04	0.02	−0.04	0.11	0.17	−0.03
*Y5 How often do you play games when you should be sleeping?*	*0.14*	*0.16*	*−0.05*	*0.18*	***0.38***	*0.29*	*0.06*	*0.02*	*0.09*	*−0.10*	*−0.03*	*0.02*
*Y15 How often do you think to yourself “just a few minutes more and then I’ll stop”?*	*0.09*	*0.03*	*0.04*	*0.22*	*0.25*	***0.37***	*−0.06*	*−0.03*	***0.32***	*0.06*	*0.06*	*0.11*
Y19 How often do you fail to meet up with a friend because you were gaming?	−0.04	−0.14	0.00	0.01	−0.05	0.07	**0.93**	**0.81**	−0.07	0.05	0.03	0.00
Y24 How often do you neglect other activities because you would rather game?	−0.03	0.06	−0.08	0.03	0.13	0.07	**0.72**	**0.80**	0.10	−0.06	−0.01	0.01
Y17 How often do you choose gaming over going out with someone?	0.07	0.13	0.05	−0.02	0.02	0.00	**0.70**	**0.79**	−0.14	−0.09	−0.04	−0.05
*Y26 How often do you lose concentration on other tasks because you are preoccupied* *with gaming?*	*0.22*	*0.15*	*0.04*	*0.11*	*0.08*	*0.03*	*0.24*	*0.18*	*0.10*	*0.18*	*0.19*	*0.25*
Y25 How often do you argue with your parents and/or your partner because of gaming?	−0.06	0.01	−0.11	−0.09	−0.01	0.00	−0.05	−0.03	**0.90**	**0.86**	0.05	0.06
Y14 How often do the people around you complain that you are gaming too much?	0.16	0.11	0.06	0.13	0.06	0.08	−0.08	−0.06	**0.63**	**0.63**	−0.06	−0.05
Y10 How often do you get restless or irritable if you are unable to play gamesfor a few days?	0.07	−0.01	−0.01	−0.05	0.03	0.10	−0.05	−0.02	0.03	−0.09	**0.78**	**0.93**
Y13 How often do you feel depressed or irritable when not gaming only for thesefeelings to disappear when you start playing?	0.09	0.06	0.02	−0.01	0.03	0.00	0.01	0.09	−0.09	0.03	**0.76**	**0.67**
Y7 How often do you get irritable, restless or anxious when you cannot play gamesas much as you want?	0.09	0.13	0.06	0.14	0.01	0.03	−0.02	−0.11	0.04	−0.05	**0.70**	**0.73**
Y21 How often do you get irritable or upset when you cannot play?	−0.11	−0.08	−0.03	−0.12	0.01	0.08	0.13	0.08	0.09	0.22	**0.68**	**0.68**

Note: Excluded items (16, 11, 2, 29, 9, 5, 15, 26) are in italic. Factor loadings ≥30 are in bold.

For the further development of this scale, items with the following rules were selected. First, items were excluded that had factor loadings lower than 0.40 at least in one of the two analyses. Second, items with salient cross loadings were excluded. If a cross loading only in one of the two parallel EFAs was identified, the cutoff 0.50 was used. In case of more than two cross-loadings, a 0.30 as a cutoff was used to exclude items from further analyses. The excluded items are crossed out in Table 2. As result of the above criteria, 18 of the original 26 items were retained (see Appendix S1).

### Confirmatory Factor Analysis

Based on the previous analyses on the samples 1 and 2, a six-factor solution was tested on Sample 3 (n = 600) with confirmatory factor analysis. This model provided an optimal fit to the data (χ2 = 256.0 df = 120 p<0.0001; CFI = 0.965; TLI = 0.956; RMSEA = 0.043 [0.036–0.051] Cfit>0.90; SRMR = 0.037). We cross-validated this model with Sample 4 (n = 1615) and found adequate level of fit (χ2 = 512.8 df = 120 p<0.0001; CFI = 0.962; TLI = 0.952; RMSEA = 0.045 [0.041–0.049] Cfit>0.90; SRMR = 0.036). The factor loadings, factor reliabilities, internal consistencies, means, and SDs are presented in Table 3.

**Table 3:** Confirmatory factor analyses of POGQ with two independent samples.

	Preoccupation		Overuse		Immersion		Social isolation		Interpersonal conflicts		Withdrawal	
	1	2	1	2	1	2	1	2	1	2	1	2
Y1 When you are not gaming, how often do you think about playing a game or thinkabout how would it feel to play at that moment?	0.87	0.84										
Y4 How often do you daydream about gaming?	0.89	0.90										
Y3 How often do you feel that you should reduce the amount of time you spend gaming?			0.76	0.72								
Y6 How often do you unsuccessfully try to reduce the timeyou spend on gaming?			0.79	0.83								
Y12 How often do you feel that gaming causes problemsfor you in your life?			0.71	0.70								
Y20 How often do you lose track of time when gaming?					0.66	0.67						
Y23 How often do you play longer than originally planned?					0.73	0.69						
Y28 How often do you feel time stops while gaming?					0.64	0.58						
Y22 How often are you so immersed in gaming that you forget to eat?					0.67	0.63						
Y19 How often do you fail to meet up with a friend because you were gaming?							0.76	0.81				
Y24 How often do you neglect other activities because you would rather game?							0.85	0.86				
Y17 How often do you choose gaming over going out with someone?							0.67	0.71				
Y25 How often do you argue with your parents and/or your partner because of gaming?									0.72	0.77		
Y14 How often do the people around you complain that you are gaming too much?									0.83	0.74		
Y10 How often do you get restless or irritable if you are unable to play games fora few days?											0.84	0.83
Y13 How often do you feel depressed or irritable when not gaming only for thesefeelings to disappear when you start playing?											0.79	0.78
Y7 How often do you get irritable, restless or anxious when you cannot play gamesas much as you want?											0.81	0.81
Y21 How often do you get irritable or upset when you cannot play?											0.77	0.76
Factor determinacies	0.94	0.94	0.91	0.91	0.91	0.90	0.93	0.93	0.90	0.90	0.95	0.94
Cronbach’s α												
Mean												
SD												

### Professional vs. Non-professional Gamers

The possible bias stemming from the inclusion of professional gamers in the total sample was checked. The level of fit of the measurement models without professional gamers (N = 2857) was satisfactory (χ2 = 763.5 df = 120 p<0.0001; CFI = 0.965; TLI = 0.956; RMSEA = 0.043 [0.040–0.046]; SRMR = 0.034). The level of fit of the measurement models only among professional gamers (N = 528) was also satisfactory (χ2 = 290.0 df = 120 p<0.0001; CFI = 0.948; TLI = 0.934; RMSEA = 0.052 [0.044–0.059]; SRMR = 0.034). Furthermore, a multi-group analysis with non-professional (N = 2857) and professional (N = 528) gamers was performed. In this analysis, the factor loadings and intercepts were set equal in both groups. The level of fit was satisfactory (χ2nonprofessional = 758.6, χ2professional = 302.4 df = 264, p<0.0001; CFI = 0.963; TLI = 0.957; RMSEA = 0.042 [0.040–0.045]; SRMR = 0.036) and the means of latent variables were not statistically different in either group.

### Labels of Factors

In the first factor, two items belonged that referred to obsessive thinking and daydreaming on the online game. This dimension was named preoccupation. The second factor contained items concerning the excessive use of online games. The three items belonging here referred to noticing gaming related problems, elongated gaming time, and the difficulties in controlling time spent on gaming. This factor was named overuse. The third factor was named immersion as these four items indicated dealing excessively with online games, immersion in gaming, and losing track of time. The fourth factor indicated damage to social relationships, and the preference of gaming over social activities. This three-item dimension was named social isolation. The two items of the fifth factor referred to the comments of the player’s social environment on overuse of online games and the related conflicts, so this factor was named interpersonal conflicts. Finally, the four items of the sixth factor concerned the appearance of withdrawal symptoms in cases when players experienced difficulties in gaming as much as they wanted. This dimension got the name withdrawal.

### Latent Profile Analysis

A latent profile analysis was performed on the dimensions of problematic online gaming, and, a four-class solution was found according to the decision criteria. As Table 4 demonstrates that the AIC, BIC and sample-size adjusted BIC continued to decrease as more latent classes were added. However, a leveling-off after the four-latent-class solution was noted. In inspection of entropy, the two-class solution reached the maximum level, but the four-class solution also provided also an adequate level of entropy. Based on the L-M-R test, the four-class solution was accepted.

**Table 4:** Fit indices for the latent profile analysis of the POGQ.

Number of latent classes	AIC	BIC	SSABIC	Entropy	L-M-R test	p
2 classes	36625	36741	36681	0.924	9567	<0.0001
3 classes	32675	32834	32752	0.909	3896	<0.0001
4 classes	30888	31090	30986	0.892	1769	<0.001
5 classes	30079	30325	30198	0.863	808	0.171

Note: AIC: Akaike Information Criteria; BIC: Bayesian Information Criteria; SSABIC: Sample size adjusted Bayesian Information Criteria. L-M-R Test: Lo-Mendell-Rubin adjusted likelihood ratio test value; p: p-value associated with L-M-R Test.

**Figure 1:**
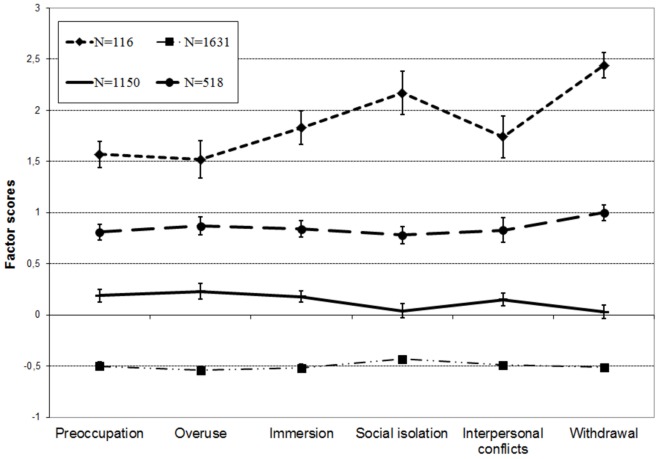
Latent profile analysis on the three factors of the POGQ . The latent profile analysis resulted four classes. The first class represents those gamers that scored on POGQ below the average, while the second class represents the low risk, the third class represents medium risk of problematic use. The fourth class (3.4%) represents the high risk of problematic use.

The features of each class are presented in Figure 1. The first class represents those gamers (47.8% of the total sample) that scored on dimensions of problematic use below the average. The second class of gamers (33.7%) represents the low risk of problematic use. The third class (15.2%) represents the medium risk of problematic use. Finally, the fourth class (3.4%) represents the high risk of problematic use. In this latter group, the ‘social isolation’ factor and ‘withdrawal symptoms’ factor especially showed an elevated level compared to other dimensions.

#### Determination cut-off score to be classified a problematic gamer: Sensitivity and specificity analyses

Based on membership in the fourth class (i.e., being at high risk for problematic gaming) as a “gold standard”, the sensitivity, specificity, PPV as well as NPV, and accuracy of the POGQ at all possible cut-off points (Table 5) were calculated.

**Table 5:** Calculation of cut-off thresholds for POGQ.

	Truepositive	Truenegative	Falsepositive	Falsenegative	Sensitivity(%)	Specificity(%)	PPV(%)	NPV(%)	Accuracy(%)
59/60	115	3179	111	0	100	97	51	100	97
60/61	115	3198	92	0	100	97	56	100	97
61/62	113	3221	69	2	98	98	62	100	98
62/63	113	3240	50	2	98	98	69	100	98
63/64	112	3257	33	3	97	99	77	100	99
64/65	111	3271	19	4	97	99	85	100	99
**65/66**	**110**	**3278**	**12**	**5**	**96**	**100**	**90**	**100**	**100**
66/67	102	3283	7	13	89	100	94	100	99
67/68	94	3285	5	21	82	100	95	99	99
68/69	88	3289	1	27	77	100	99	99	99
69/70	79	3289	1	36	69	100	99	99	99
70/71	68	3289	1	47	59	100	99	99	99
71/72	65	3289	1	50	57	100	98	99	99
72/73	54	3290	0	61	47	100	100	98	98
73/74	47	3290	0	68	41	100	100	98	98

Based on this analysis, a cut-off score of 65 points is suggested as an ideal cut-off to be classed as problematic gamer. In this case, specificity is 100%, while sensitivity is 96%. This means that practically none of the negative (i.e., non-problematic) cases are considered as problematic, while only 4% of the true problematic cases are not recognized. Accuracy, as well as NPV in this case is 100%, while PPV is 90%. Increasing of the cut-off score would result in the growing number of the false negative cases, whereas decreasing would lead to more false positive cases.

## Discussion

Due to the growing number of indicated problems concerning online gaming it has become an absolute necessity to develop a tool with adequate psychometric characteristics for the measurement of the extent of gaming-related problems. The Problematic Online Gaming Questionnaire (POGQ) developed in this study, based on the results of the analyses, appears to fulfill those requirements that are expected from a measure like this. The POGQ was created in a way that it is applicable for all types of online games and its empirical basis makes it possible to cover all problems experienced by the players.

The results of these empirically-based analyses are at the same time very much supported by the fact that the six dimensions identified in the background of problematic online gaming fit closely to the available theoretical frameworks. Griffiths [18] proposed a “components” model for addictions that assumes the six classical symptoms for addiction behaviors in general that is salience, mood modification, tolerance, withdrawal, conflict, and relapse. The withdrawal and preoccupation components can be identified with our equally named factors, while conflict dimension is partly covered by the interpersonal conflicts factor and partly by the factor overuse (intrapersonal conflicts). It is interesting though that items explicitly representing salience dimension fell out during analysis (see item 2, 5, 11 in Table 2.), but the component is still present in overuse, preoccupation, and social isolation factors. The relapse component appears in the overuse dimension while mood modification dimension is primarily present in the withdrawal factor (item 13 in Table 2).

In another approach, the DSM-IV criteria for psychoactive substance use dependence by the American Psychiatric Association [29] that are generally regarded as the definitional basis of behavioral addictions can be considered. These dimensions – withdrawal, lack of control (unsuccessful attempt to quit), much time spent on the activity, behavior continues despite knowledge of adverse consequences, more intensive use and for longer period than intended – are all clearly reflected in the POGQ. The more than adequate psychometric properties of the POGQ and the wide empirical content it is based on, is reassuring regarding the future use of the scale. However, further tasks include the cross-cultural validation of the POGQ and clinical validation of the scale.

It is an issue in relation to all behavioral addictions not present in DSM-IV-TR whether those individuals who engage in a specific behavior excessively should be regarded as having a disorder, and with which criteria we should identify individuals functioning on other pathologic levels. Considering the lack of consensus regarding definitions, the authors of the present study insisted on using the expression ‘problematic gaming’ instead of the more ambiguous gaming dependence. However, the latent profile analysis performed also indicated that a segment of the online gaming population (3.4% in this study) significantly exceeded the whole population and characteristically showed more problems than others. A further 15.2% of the population also showed moderately elevated level of problems. One of the most important tasks in future research is the detailed analysis of this at-risk population and to explore which background factors may carry high risk concerning problematic gaming. For forthcoming studies, the results here highlight two significant dimensions, withdrawal and social isolation, that showed elevated levels in case of these gamers, while obsession and overuse seemed to be the less indicative dimensions. Therefore, it seems that intensive actual (overuse) or imaginary (obsessive) gaming is less indicative of problematic gaming in itself. These results coincide with the results observed concerning problematic internet use [30]. In contrast, neglecting social relationships and especially the presence of withdrawal symptoms (feeling depressed or irritable, getting restless, anxious or upset when not able to game) appear to carry the highest risks. Furthermore, it is important to note that both dimensions include reducing or neglecting other activities which are key characteristics of addictions according to the results of many other studies.

One limitation of the present study is that it was carried out among Hungarian gamers thus results should be cautiously generalized for other cultures. However, it is hoped that future studies can confirm the findings presented here in other cultures. Another important issue is that current results were based on self-report data. It is again a challenge for future studies to investigate and confirm the identified problem-dimensions in clinical and/or observational studies. In conclusion, and based on all these assumptions, it is hoped that creation of the POGQ will facilitate and enhance further research, and that the instrument will serve as a valid and reliable tool in future studies.

## Supporting Information

Appendix S1 **Problematic Online Gaming Questionnaire (POGQ).**
(DOC)
