# Supplementary material for: The Development of the Problematic Online Gaming Questionnaire (POGQ)
Source: PLoS One. 2012 May 10;7(5):e36417. doi: 10.1371/journal.pone.0036417 (PMC3349662; doi:10.1371/journal.pone.0036417)
Supplement: Appendix S1 — Problematic Online Gaming Questionnaire (POGQ). (DOC) [file pone.0036417.s001.doc]

**Appendix S1**

**Problematic Online Gaming Questionnaire (POGQ)**

Please read the statements below regarding *online gaming*. The questionnaire REFERS TO ONLINE GAMES exclusively, but we use the expression ’game’ in each statement for simplicity’s sake.

Please indicate on the scale from 1 to 5 to what extent, and how often, these statements apply to you!

|  | never | seldom | occasionally | often | always |
| --- | --- | --- | --- | --- | --- |
| 1. When you are not gaming, how often do you think about playing a game or think about how would it feel to play at that moment? | **1** | **2** | **3** | **4** | **5** |
| 2. How often do you play longer than originally planned? | **1** | **2** | **3** | **4** | **5** |
| 3. How often do you feel depressed or irritable when not gaming only for these feelings to disappear when you start playing? | **1** | **2** | **3** | **4** | **5** |
| 4. How often do you feel that you should reduce the amount of time you spend gaming? | **1** | **2** | **3** | **4** | **5** |
| 5. How often do the people around you complain that you are gaming too much? | **1** | **2** | **3** | **4** | **5** |
| 6. How often do you fail to meet up with a friend because you were gaming? | **1** | **2** | **3** | **4** | **5** |
| 7. How often do you daydream about gaming? | **1** | **2** | **3** | **4** | **5** |
| 8. How often do you lose track of time when gaming? | **1** | **2** | **3** | **4** | **5** |
| 9. How often do you get irritable, restless or anxious when you cannot play games as much as you want? | **1** | **2** | **3** | **4** | **5** |
| 10. How often do you unsuccessfully try to reduce the time you spend on gaming? | **1** | **2** | **3** | **4** | **5** |
| 11. How often do you argue with your parents and/or your partner because of gaming? | **1** | **2** | **3** | **4** | **5** |
| 12. How often do you neglect other activities because you would rather game? | **1** | **2** | **3** | **4** | **5** |
| 13. How often do you feel time stops while gaming? | **1** | **2** | **3** | **4** | **5** |
| 14. How often do you get restless or irritable if you are unable to play games for a few days? | **1** | **2** | **3** | **4** | **5** |
| 15. How often do you feel that gaming causes problems for you in your life? | **1** | **2** | **3** | **4** | **5** |
| 16. How often do you choose gaming over going out with someone? | **1** | **2** | **3** | **4** | **5** |
| 17. How often are you so immersed in gaming that you forget to eat? | **1** | **2** | **3** | **4** | **5** |
| 18. How often do you get irritable or upset when you cannot play? | **1** | **2** | **3** | **4** | **5** |

| preoccupation | immersion | withdrawal | overuse | interpersonal conflicts | social isolation |
| --- | --- | --- | --- | --- | --- |
| **1, 7** | **2, 8, 13, 17** | **3, 9, 14, 18** | **4, 10, 15** | **5, 11** | **6, 12, 16** |
